# Oral pharmacokinetics of a pharmaceutical preparation of florfenicol in broiler chickens

**DOI:** 10.3389/fvets.2023.1208221

**Published:** 2023-06-07

**Authors:** Lilia Gutierrez, Aline Guzman-Flores, Minerva Monroy-Barreto, Luis Ocampo, Hector Sumano

**Affiliations:** ^1^Departamento de Fisiología y Farmacologia, Faculta de Medicina Veterinaria y Zootecnia, Universidad Nacional Autónoma de México, Ciudad de México, Mexico; ^2^Departamento de Química Analítica, Facultad de Química, Universidad Nacional Autónoma de México, Ciudad de México, Mexico

**Keywords:** florfenicol-premix, florfenicol-FOLA, broiler-chicken, pharmacokinetics, oral dosage

## Abstract

**Introduction:**

The use of florfenicol must follow particular pharmacokinetic/pharmacodynamic (PK/PD) ratios, i.e., it requires achieving serum concentrations at or slightly above the pathogen's minimum inhibitory concentration (MIC) during the dosing interval and that the ratio of area under the concentration vs. time curve (AUC)/MIC should be as high as possible (still undetermined for poultry). As an alternative to the standard soluble florfenicol that is administered to the flock through drinking water, florfenicol premix is often recommended as feed medication in Latin America. However, no particular pharmaceutical design has been proposed.

**Methods:**

This study compared the PK of two preparations of florfenicol in broiler chickens and pondered the possibility of each covering the referred PK-PD ratios as predictors of clinical efficacy. The preparations comprise a pharmaceutical form as FOLA pellets (F = bioavailability; O = optimum; and LA = long-acting) and the premix formulation. The former are small colored pellets with vehicles and absorption enhancers of florfenicol designed for long action, and the latter is the reference premix of the antibiotic. First, these two pharmaceutical forms of florfenicol were administered as oral boluses (30 mg/kg), aided by a probe. In a second trial of the dosing form, both pharmaceutical preparations of florfenicol were administered in feed and ad libitum (110 ppm; ~30 mg/kg).

**Results:**

In both cases, FOLA-florfenicol presented much higher relative bioavailability (3.27 times higher) and mean better residence time than florfenicol premix (two times high when forced as bolus dose). Consequently, FOLA-florfenicol possesses better PK/PD ratios than less sensitive pathogens, i.e., *E. coli*. It is proposed that if a metaphylactic treatment of a bacterial outbreak in poultry is implemented with florfenicol prepared as FOLA, better PK/PD ratios will be obtained than those of standard florfenicol premix.

**Discussion:**

Clinicians must confirm that feed consumption in the flock has not been affected by the particular disease if FOLA pellets of florfenicol are used.

## Introduction

Florfenicol is a wide-spectrum, synthetic antibacterial structurally related to chloramphenicol and thiamphenicol. To date, there is no evidence of toxicity or relevant adverse effects in poultry for this derivative. In contrast, chloramphenicol has been banned in most countries due to its involvement in human toxicity, i.e., aplastic anemia that can be developed even with residual amounts (1). The recommended dose in poultry is 30–40 mg/kg bw/day for 3 days *via* drinking water, and its pharmacokinetics has been defined for this vehicle (2, 3). In contrast, no published data recommend its use as an in-feed medication in poultry species. Nevertheless, several florfenicol premix preparations are available for poultry in Latin American countries. However, in broiler chickens, the oral bioavailability appears to be lower in fed (55%) than in fasted animals (87–96%) (3, 4). This feature may indicate that administering florfenicol in poultry as an in-feed medication may or may not achieve adequate serum and tissue concentrations and consequently may or may not deliver good clinical efficacy. Florfenicol shows an elimination half-life of at least 106.6 min in poultry when administered through their drinking water (5, 6), and it exhibits a reasonably good apparent volume of distribution at a steady state (3.5 ml/kg) (6). When experimentally administered by an oral gavage at a dose of 40 mg/kg bw, the duration of therapeutic plasma concentrations can be stretched up to 8 h (7). If administered *ad libitum* through the drinking water (approximate dose of 26 mg/kg bw) in an 18:6 dark:light cycle for 3 days, the estimated mean serum concentration of florfenicol averaged ~ 0.7 μg/ml, and no florfenicol was detected in serum 72 h after the terminal dose. Florfenicol is partially metabolized into florfenicol amine, which is still bioactive (3).

Florfenicol is usually prescribed for treating gastrointestinal and respiratory tract infections in poultry. The presence of a bacterial disease must be established to administer florfenicol as a metaphylaxis treatment (8). Based on the above features of florfenicol, it is postulated that it could be administered in poultry as feed medication and achieve good bioavailability in broiler chickens. According to Murugayan et al. (8), it is necessary to seek alternatives in dosing antibacterial drugs to chicken to optimize pharmacokinetic/pharmacodynamic ratios and reduce the emergence of bacterial resistance caused by the misuse of these drugs. This trial was set to test this hypothesis; that is, a comparative pharmacokinetic study was carried out in broiler chickens, evaluating an experimental long-acting dosage form of florfenicol, prepared as small colored pellets, and named FOLA [Patent No. MX/a/2012/013222 and PCT/MX2013/000137, Universidad Nacional Autónoma de México [UNAM]; FOLA stands for bioavailability (F), optimum (O), and long-acting preparation (LA)]. A standard commercial premix of florfenicol for in-feed administration was taken as a reference preparation.

## Materials and methods

The study design and animal handling complied with Mexican regulations for experimental animals, as stated by CICUA No. 0673 (Internal Committee for the Care and Use of Animals, UNAM) and Mexican prescripts in NOM-062-ZOO-1999. This trial was carried out in an experimental chicken house in Mexico City. Overall, 360 15-day-old Ross-308 broiler chickens, weighing approximately 450 g with a daily gain of 60 g, were distributed by simple randomization in four groups. They were allocated within a single chicken house. It was divided by wire mesh into eight smaller areas to contain 45 chicken broilers per group, i.e., a group and its replica as follows: for bolus dose, (F_REF − bolus_) florfenicol premix from the product NF-180^®^ 8% (PiSA Agropecuaria S.A. de CV, Mexico; https://www.avicultura.mx/producto/nf-180-8) approved in Mexico and other Latin American countries for poultry.

The bolus administration of the florfenicol premix or the florfenicol FOLA was carried out at 6 a.m. Each pharmaceutical preparation was weighed to meet individual broiler chicken weights. Each preparation was suspended in tap water with 2% gelatin and stirred prior to its dosing. The administration was achieved by employing a plastic probe attached to a 10 ml syringe and introduced into the included samples. In both groups, the established dose was 30 mg/kg (15 mg/chicken broiler). Once ensured that no regurgitation occurred, broiler chickens were allowed to feed and water *ad libitum*. For the *ad libitum* dosing (F_REFad − lib_ and F_FOLAad − lib_), florfenicol, either as premix from NF-180^®^ 8% or FOLAs, was incorporated into their powdered feed at a rate of 110 ppm of florfenicol, considering a feed intake of 140 g/chicken and a final dose of ~ 30 mg/kg/day (15 mg/chicken, considering a 10% feed-waste). Medicated feeds were prepared daily. In addition, FOLAs were added to the powdered feed as a dressing at the same dose rate, establishing visually that the distribution of pellets was even. In both *ad libitum* groups, florfenicol was administered for 3 days, making feeders available from 6:00 a.m. to 12:00 p.m. during these days. Diet composition is presented in Table 1.

**Table 1:** Diet composition for this trial with 15-day-old Ross-308 broiler chickens, weighing ~ 450 g.

**Ingredients**	**Amount (kg)**
Corn	590.50
Soybean meal	344.80
Soybean oil	23.80
Salt	2.40
Calcite limestone	9.50
Dicalcium phosphate	18.00
DL-methionine	1.75
L-lysine	2.15
Vitamins^a^	2.50
Minerals^b^	1
Sodium bicarbonate	3.60
Total	1,000
Crude protein	207.90
Calcium	8.80
Available phosphorus	4.40
Methionine	4.90
Sulfur amino acids	8.20
Lysine	12.70
Potassium	8.00
Sodium	2.20
Chlorine	1.90
Linoleic acid	26.00
Metabolizable energy, MJ/kg	12.56

a Amount/kg: Retinol 0.9 g, cholecalciferol 0.019 g, d-alpha-tocopherol 0.004 g, phylloquinone 1.0 g, riboflavin 4.0 g, cyanocobalamin 0.060 g, pyridoxine 3.0 g, calcium pantothenate 13.0 g, niacin 25 g, biotin 0.063 g, and choline chloride 250 g.

b Amount/kg: selenium 0.2 g, cobalt 0.1 g, iodine 0.3g, copper 10 g, zinc 50 g, iron 100 g, and manganese 100 g.

The pharmacokinetic approach of this study was to obtain PK parameters following a type of naïve pooled sampling since each animal was not sampled more than two times. Hence, for the oral bolus dosing (F_REF − bolus_ and F_FOLA − bolus_) of this trial aided by technical assistance, ~ 1 ml of blood samples was obtained by jugular or radial wing vein puncture with 3 ml syringes and 25g x 1 in pediatric needles. A total of 10 broiler chickens were bled-sampled each time, i.e., 5 from each group and 5 from each repetition. The set times were as follows: 0.5, 1, 2, 4, 6, 8, 12, and 24 h. No bird was sampled more than twice in a 24-h period.

For the *ad libitum* dosing, blood samples (1 ml per chicken) were obtained from 5 animals in each group and 5 from its repetition during 3 days of medication at fixed times as follows: 2, 4, 8, 14, and 24 h after dosing, i.e., 8 a.m., 10 a.m., 2 p.m., 8 p.m., and 6 a.m. the next morning and on each day. Paint marking of the sampled broiler chickens allowed for an even sampling in each group. Blood samples were centrifuged at 1,000 × *g* for 5 min, and serum was harvested and stored at −20°C until analyzed.

The FOLA pellets of florfenicol were manufactured in our laboratory as described in Patent No.MX/a/2012/013222 and CT/MX2013/000137 (UNAM), observing good laboratory practices. In brief, 1% carpool with butylhydroxytoluene as an antioxidant was mixed in a base of 1:1 parts of wheat and corn flour. Then, a yellow-orange vegetable dye was added. Finally, florfenicol was incorporated at a rate of 10%. The mixture was mixed and then extruded at temperatures not higher than 30°C, using ethylalcohol and cotton-seed oil as lubricants. The final concentration of florfenicol in FOLAS was 97.6%, as determined by high-performance liquid chromatography (HPLC) based on the established technique (10).

Concentrations of florfenicol and its active metabolite florfenicol-amine were determined in plasma samples by HPLC using the method described by Kowalski et al. (11), with thiamphenicol as an internal standard. In brief, the extraction procedure was initiated by thawing the plasma samples at 20–25°C laboratory temperature. Then, 0.5 ml of plasma aliquot thiamphenicol was added as internal standard (0.5 μg in 0.2 ml), along with 0.2 ml of 1.0 M sodium hydroxide and 3 ml of ethyl acetate. Each sample was vortex mixed and centrifuged at 5,000 *g* for 15 min, and the organic layer was carefully transferred to another tube. The supernatant was dissolved in 0.5 ml of the mobile phase. Then, the samples were filtered through a membrane (nylon 0.45 μm) and injected into the HPLC with a 0.6 ml/min flow. Acetonitrile–water (25:75, v/v), adjusted to a pH of 2.7 with 85% orthophosphoric acid, was utilized as the mobile phase. Detection and quantitation were performed at 224 nm for excitation wavelength and 290 nm for emission wavelength. Calibration curves for florfenicol and florfenicol-amine were prepared from 0.05 to 20.48 μg/ml (*n* = 5).

The apparatus used was a Jasco XLC HPLC system (LC-2000Plus; Jasco Benelux, the Netherlands) with a Symmetry-C18 column (4.6 mm × 100 mm, 3.5 μm; Waters, USA) and equipped with a fluorescence detector. Data were analyzed using Empower-3 software from Waters (Mexico). The chromatographic method was validated, and the analytical procedure was demonstrated as specific. The method produced a linear result from 0.05 to 20.48 μg/ml (r^2^ = 0.984; y = 500030 x−107 046). Recovery of florfenicol and florfenicol-amine was calculated by applying a linear regression analysis. Precision was demonstrated by the inter-day coefficient of variance (3.0) and the inter-assay error value (< 3.8). The lower quantification limit for florfenicol in plasma was 0.05 μg/ml, with a detection limit of 0.008 μg/ml, and linearity was established from 0.05 to 20.48 μg/ml.

A pharmacokinetic analysis of plasma concentration–time data for florfenicol was carried out using PKAnalyst^®^ (Micromath, Scientific Software, SLM, USA). The pharmacokinetic model (11) was based on choosing the most similar one having the highest r after examining the concentration–time curves (*r* > 0.98). Then, the number of exponential terms required to describe the plasma concentration–time data for each dosing form was determined by applying Akaike's information criterion (12). The peak concentration in serum (C_MAX_) and the time to C_MAX_ (T_MAX_) were estimated by observing data from tabulated plasma concentrations. Relative bioavailability (Fr%) was derived by comparing AUC_0 − 24_ with florfenicol-FOLA and florfenicol-reference groups.

The serum concentration *vs*. time data was graphed with Origin Lab-Pro 8C. Areas under the concentration *vs*. time curve (AUC) on days 1 and 3 were calculated through the trapezoidal method and confirmed with PKAnalyst^®^. Statistical AUC values were compared with ANOVA and successive Dunnet's test.

## Results

**Figure 1:**
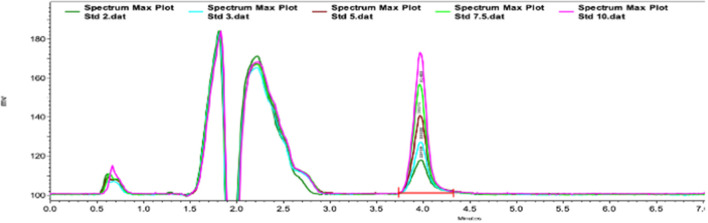
Type chromatogram of florfenicol in chicken serum samples. The retention time for florfenicol was 4 min.

**Figure 2:**
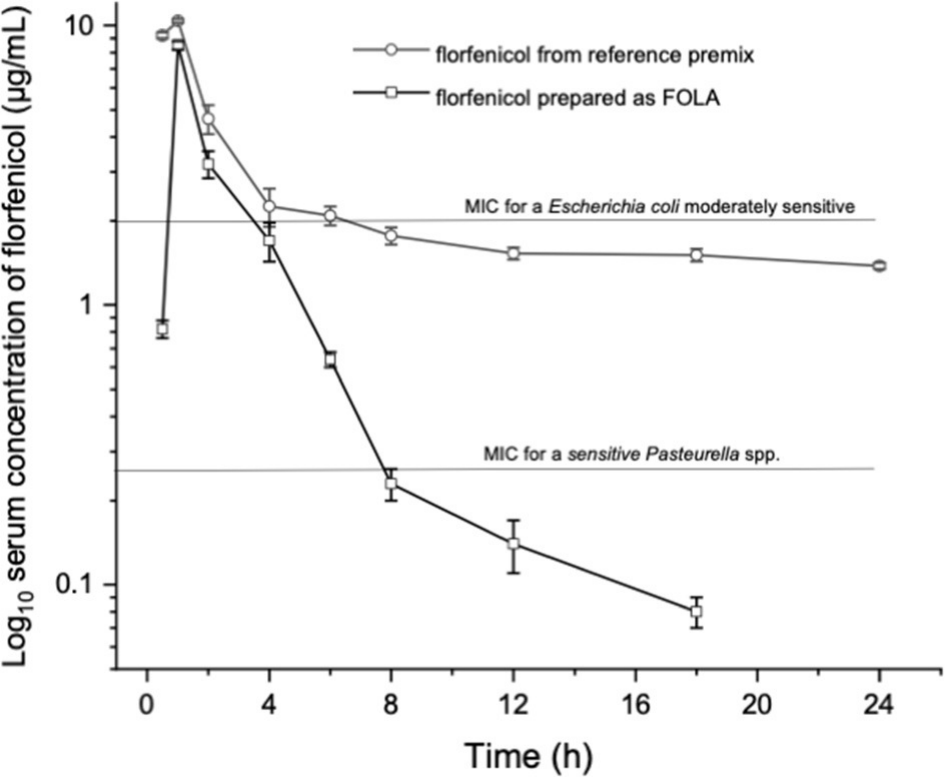
Florfenicol serum concentrations in broiler chickens treated with 30 mg/kg florfenicol either as a reference premix (F_REFbolus_) or as FOLA pellets (F_FOLAbolus_), employing a probe and suspending either preparation in water plus 2% gelatin.

**Figure 3:**
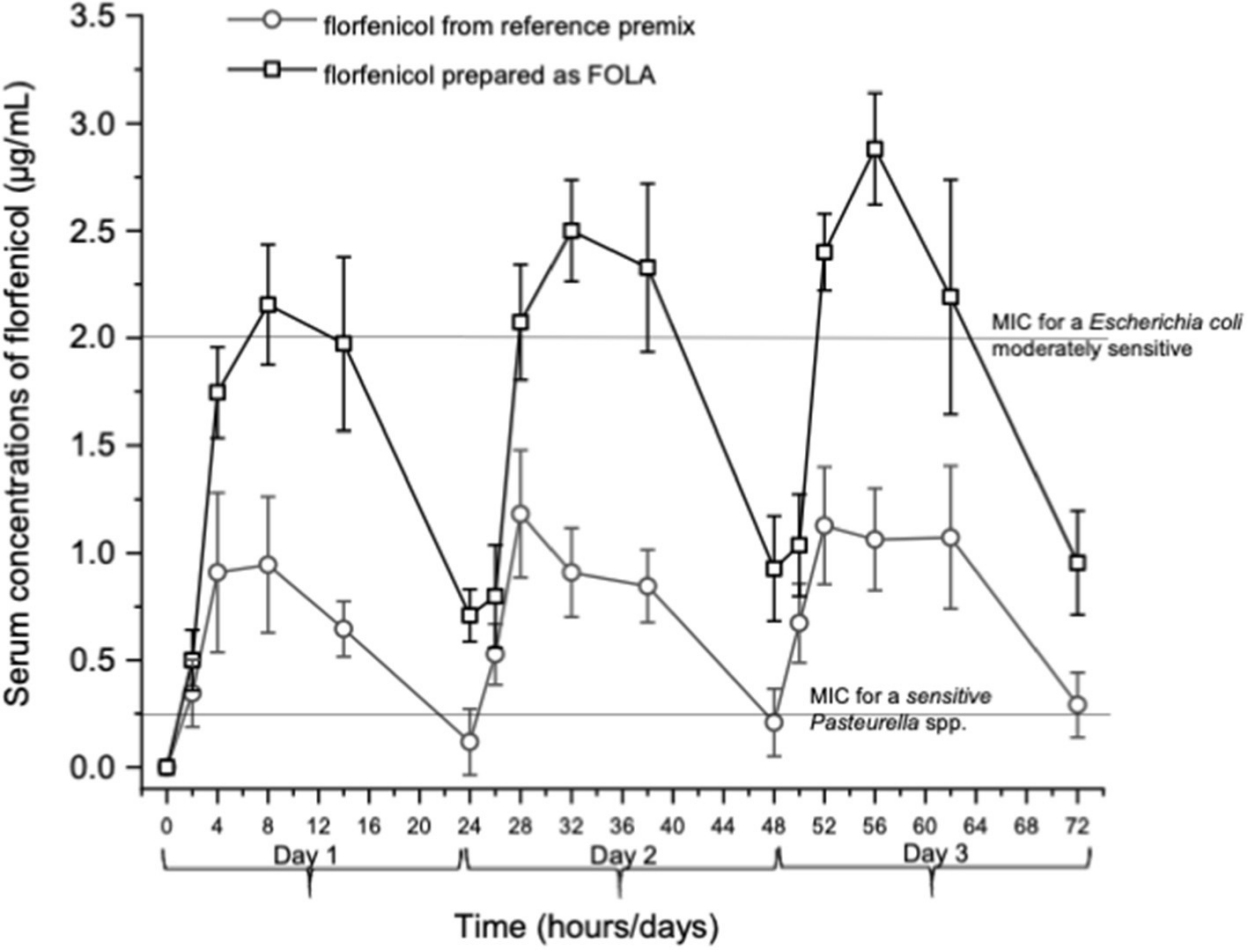
Serum concentrations of florfenicol in broiler chickens receiving two pharmaceutical forms of florfenicol. Feed medication was made available to chickens at 6:00 a.m. using powdered premix (NF-180^®^, PiSA Agropecuaria S.A. de CV, Mexico) or small colored pellets (FOLA-see text). In both instances, 110 ppm of florfenicol was incorporated into the feed. Given a food intake of 140 g/chicken (weighing 450 g), a dose of ~30 mg/kg was administered.

Figure 1 shows an example of the chromatograms obtained. The method utilized showed linearity when florfenicol-fortified chicken serum samples were analyzed. Mean ± 1 SD serum concentrations *vs*. time profiles of florfenicol in broiler chickens after forced oral bolus medication with either florfenicol premix from NF-180^®^ or florfenicol prepared as FOLAs are presented in Figure 2. Figure 3 shows the serum concentrations of florfenicol after its administration *ad libitum* (110 ppm) for 3 days and their pharmacokinetic variables are shown in Table 2. Pharmacokinetic data obtained for oral bolus administration are shown in Table 3. In addition, two pharmacokinetic/pharmacodynamic (PK/PD) ratios (AUC_0 − 24_/MIC and %T ≥ MIC) are presented for two pathogenic bacteria whose MIC values were taken from the formal literature: one sensitive (0.25 μg/ml) and the other moderately sensitive (2.0 μg/ml) (13, 14). The relative bioavailability value of F_FOLA − bolus_ was 328%. Comparisons for MRT and T$\frac{1}{2}$β showed that these variables were statistically longer (*P* < 0.05) in F_FOLA − bolus_ compared with F_REF − bolus_. The time the serum florfenicol levels remain above or equal to MIC values (T≥MIC) and the AUC_0 − 24_/MIC ratios for F_FOLA − bolus_ show that the latter had larger ratios than the former. Florfenicol administered as premix remained above MIC values of the theoretically sensitive bacteria (0.25 μg/ml) 31% of the dosing interval (24 h), while florfenicol prepared as FOLA was capable of covering the whole dosing interval. However, the same T ≥ MIC ratio is reduced to 16% and 30% for F_REF − bolus_ and F_FOLA − bolus_, respectively, if the challenged bacteria are moderately sensitive (2.0 μg/ml). AUC_0 − 24_/CMI ratios follow the same pattern favoring the FOLA form of florfenicol.

**Table 2:** Mean ± 1 SD pharmacokinetic parameters of florfenicol in broiler chickens receiving two pharmaceutical forms of florfenicol as in-feed medication and *ad libitum*: powdered premix (NF-180^®^, PiSA Agropecuaria S.A. de CV, Mexico) (F_REFad − lib)_, and as FOLA (F_FOLAad − lib)_.

**Parameter**	**F**_**FOLAad − lib**_		**F**_**red − lib**_	
	**Mean**	±**1 SD**	**Mean**	±**1 SD**
Ka (h^−1^)	0.12^a^	0.6	0.6 ^a^	0.3
T$\frac{1}{2}$β_1_ (h)	3.17 ^a^	0.2	1.8 ^a^	0.8
T $\frac{1}{2}$ β_2_	6.2^a^	0.3	2.3 ^a^	0.4
T $\frac{1}{2}$ β_3_	7.0 ^a^	0.6	4.2 ^b^	1.2
AR_Cmax_	2.33	0.2	1.42	0.2
AR_AUC_	1.13	0.2	1.09	0.1
Fr (%)	250.6			
AUC_0 − 72_ (μg/mL·h)	132.6^a^	6.5	52.9^b^	4.9
AUC_0 − 24_ (μg/mL·h)	44.0^a^	2.5	17.6^b^	1.8
AUC_T_ (μg/mL·h)	137.2^a^	8.5	53.7^b^	6.2
MRT_1_ (h)	16.9^a^	3.2	14.1^b^	2.1
RT_1_ (h)	16.9	3.2	14.1	2.1
RT_lastT_ (h)	16.8	3.4	14.0	2.4
AUC_24 − 48_ (μg/mL·h)	17.95	2.67	46.24	5.31
AUC_48 − 72_ (μg/mL·h)	18.92	3.65	46.90	5.28
AUMC_1_ (μg/mL·h^2^)	745.9	13.5	248.0	9.14
AUMC_lastT_ (μg/mL·h^2^)	739.5	14.1	247.3	10.1

Ka, absorption rate constant; T$\frac{1}{2}$β_1, 2, 3_, elimination half-life on days 1, 2, and 3; Fr, relative bioavailability (F_FOLAad − lib_/F_REF_ x 100); AUC_0 − 72_, area under the curve from 0 to 72 h; ^st^AUC, first-day area under the curve from 0 to 24 h; 1^st^AUC_T_, first-day area under the curve-trapezoidal method; RT_1_, Residence time at first dose; RT_lastT_, Residence time at last T; AR_Cmax_, Accumulation ratio using C_max_ as a comparative value; AR_AUC_, Accumulation ratio using AUC_0 − 24_ as a comparative value; AUMC_1_, area under the moment curve at first dose; AUMC_last_ T, area under the moment curve at last T.

In both instances, 110 ppm of florfenicol were incorporated into feed, which, given a food intake of 140 g/chicken (weighing 450 g), a 30 mg/kg dose was calculated.

a, b Different letters mean statistical differences between groups (*P* < 0.5).

**Table 3:** Mean ± 1 SD of the oral pharmacokinetic parameters of florfenicol in broiler chickens after a 30 mg/kg administration either as a reference premix (F_REFbolus_) or as FOLA pellets (F_FOLAbolus_), utilizing a probe and suspending either preparation in water plus 2% gelatin.

**Parameter**	**F**_**REFbolus**_		**F**_**FOLAbolus**_	
	**Mean**	±**1 SD**	**Mean**	±**1 SD**
Ka (h^−1^)	0.86 ^a^	0.04	0.23^b^	0.04
T$\frac{1}{2}$β (h)	2.35^a^	0.31	8.72^b^	0.48
T_MAX_ (h)	1.23^a^	0.31	1.07^a^	0.01
C_MAX_ (μg/mL)	7.17^a^	0.52	10.31^b^	1.25
AUC_0 − 24_ (μg/mL·h)	17.25^a^	2.42	41.18^b^	5.1
AUC_0−∞_ (μg/mL·h^−2^)	42.32^a^	5.74	162.01^b^	13.68
MRT (h)	3.45^a^	0.71	7.03^b^	0.45
AUC_T_ (μg/mL·h)	18.14^a^	2.32	59.62^b^	6.61
F %	100%		328.7	
**PK/PD ratios**				
AUC_0 − 24_/CMI${}_{0.25}^{*}$	69		165	
%T ≥ CMI${}_{0.25}^{*}$	31%		>100%	
AUC_0 − 24_/CMI${}_{2.0}^{\text{\#}}$	8.6		20	
%T ≥ CMI${}_{2.0}^{\text{\#}}$	16%		30%	

Ka, absorption rate constant; K$\frac{1}{2}$_ab_, absorption constant; C_MAX_, Maximum plasma concentration; T_MAX_, time at which C_MAX_ is achieved; AUC_0 − 24_, area under the curve from 0 to 24 h; MRT, mean residence time; AUC_0−∞_, area under the curve from zero to infinity; AUC_T_, area under the curve-trapezoidal method; Fr, relative bioavailability (F_FOLAbolus_/F_REFbolus_ x 100); PK/PD ratios: AUC_0 − 24_/CMI_0.25_> 125; %T ≥ CMI_2.0_ > 100%.

^*^Pasteurella spp. sensitive 0.25 μg/mL.

^#^*Escherichia coli* moderately sensitive 2.0 μg/mL.

Relevant PK/PD ratios are also presented.

a, b Different letters mean statistical differences between groups (*P* < 0.5).

In the *ad libitum* administration, a decisive difference in the serum profiles of florfenicol for the FOLA form of florfenicol is appreciated. The values of AUC_0 − 72_ and 1^st^AUC_0 − 24_ and 1st MRT and T$\frac{1}{2}$β were statistically higher than those achieved with the reference florfenicol premix (*P* < 0.05 in all cases). Consequently, this complies well with a lower Ka obtained for F_FOLAbolus_ and F_FOLAad − lib._

## Discussion

Respiratory diseases are the primary problem in poultry production worldwide, and although much can be done with good husbandry, bacterial disease outbreaks occur and must be treated (15, 16). Antimicrobial drugs are then chosen and are critical to solving the problem. However, their utility has been compromised in recent years by the emergence of resistance to antimicrobial drugs. Given the increasingly limited availability of antimicrobial drugs for poultry production, it is reasonable to think that an immediate operational line of research is to optimize the pharmaceutical design of each active principle (9). The study on the absorption and bioavailability processes of antibacterial drugs in poultry is limited (17). Most pharmaceutical forms available for poultry medicine have been the product of trial and error. They were not designed to optimize their PK/PD ratios when included in food or drinking water (18). Thus, when an outbreak of a respiratory disease occurs, such as complicated chronic respiratory disease, infectious coryza (*Haemophilus paragallinarum*), or fowl cholera (*Pasteurella multocida*), soluble florfenicol is often administered as the drug of choice. Early intervention is required (metaphylaxis), and it has been postulated that sick birds tend to reduce feed or water intake, but their consumption patterns before signs of the referred diseases appear have yet to be established; that is, unless a critical part of the flock is affected, water or feed intake variations are rarely detected during the early stages of the disease. This is a task that requires careful research. However, in this context, several preparations of florfenicol in premix became available in Latin America for metaphylaxis treatment.

Florfenicol is rapidly eliminated from the broiler chickens' plasma. This study conceived and tested an attempt to extend the clearance of florfenicol with its inclusion in FOLA pellets and as an in-feed medication. The results show improved florfenicol PK/PD ratios. It is postulated that the gastrointestinal retentive properties of carbopol in FOLA pellets modify absorption into a type of sustained release, and therefore, elimination is extended.

Prudent use of highly potent antimicrobial drugs in veterinary medicine, such as florfenicol, is required (9, 17). To achieve this goal, it is necessary to design pharmaceutical preparations that better comply with each drug's competent PK/PD ratios (19). In turn, this may contribute to maintaining its efficacy in the future (10, 20, 21). It is in this context that florfenicol in FOLAs was conceived. FOLAs can be described as pharmaceutically designed carriers prepared as small color pellets that are readily consumed by poultry, which allow the inclusion of vehicles and help improve the absorption of the active ingredient by modulating the GI-transit time (10, 21).

The pharmacokinetic parameters obtained for the reference florfenicol as premix were very similar to what was achieved in other studies with soluble florfenicol. Minor differences can be attributed to the use of different bloodlines of chicken, different ages, feeding, and housing peculiarities (22, 23). More efficient absorption of florfenicol, when prepared as FOLA pellets, was anticipated as sustained-release formulations tend to achieve better bioavailability values (20, 24), and such behavior has already been obtained with FOLAs for doxycycline and tylosin in poultry (19, 23). Furthermore, the lower Ka obtained for F_FOLAbolus_ and F_FOLAad − lib_ may suggest that a flip-flop phenomenon is occurring. In this study, the F_FOLAbolus_ preparation achieved better values in C_MAX_ and AUC_0 − 24_ than the premix formulation. In addition, MRT and T$\frac{1}{2}$β were statistically higher than the premix formulation (*P* < 0.05). Consequently, these features generate notable differences in the bioavailability of the preparations, with a notable advantage for the pharmaceutical form of type FOLA, i.e., Fr from 250 to 328%. Having higher plasma concentrations of the antibacterial drug and a total load of florfenicol achieved (AUC/CMI), as well as slower elimination parameters for florfenicol FOLA compared with reference florfenicol premix (T ≥ CMI), signify a notable improvement in the pharmacokinetic ratios of this drug. Florfenicol is considered a time-dependent antibacterial, and it would seem that there is no advantage in using FOLAs when treating sensitive bacteria (MIC = 0.25 μg/ml). However, it is evident that if a more resistant pathogen is involved in an outbreak, such as *E. coli* (24), only florfenicol prepared as FOLA will achieve adequate PK/PD ratios. This latter view is even more evident in the *ad libitum* dosing, where the AUC values, T$\frac{1}{2}$B, and MRT parameters were substantially higher.

In summary, during the metaphylactic treatment of a bacterial outbreak in poultry, the clinician can safely use florfenicol prepared as FOLA, as better PK/PD ratios than those achieved with florfenicol premix will be obtained. In turn, better clinical results are foreseen. These pharmacokinetic considerations apply if the clinician can confirm that feed consumption in the flock has not been affected by the particular disease. In addition, given the prolonged MRT, AR_Cmax_, and AR_AUC_ found for FOLA preparations, a new withdrawal time may be necessary to avoid unauthorized drug residues. Nevertheless, according to the standards proposed by the European Medicines Agency for the modified release of pharmaceutical preparations, the obtained value for the FOLA pellets (AR_AUC_ < 1.25) can be considered as leading to non-relevant drug accumulation (24).

## Data availability statement

The raw data supporting the conclusions of this article will be made available by the authors, without undue reservation.

## Ethics statement

The animal study was reviewed and approved by CICUA No. 0673 (Internal Committee for the Care and Use of Animals, UNAM) and Mexican prescripts in NOM-062-ZOO-1999.

## Author contributions

HS and LG conceived and designed the study and carried out the pharmacokinetic and statistical analyses. AG-F and LO carried out the clinical trial. MM-B and AG-F performed the analytical phase. All authors have read and accepted the manuscript as it is presented to the journal.
